# Tetrapod-like pelvic girdle in a walking cavefish

**DOI:** 10.1038/srep23711

**Published:** 2016-03-24

**Authors:** Brooke E. Flammang, Apinun Suvarnaraksha, Julie Markiewicz, Daphne Soares

**Affiliations:** 1 Department of Biological Sciences, New Jersey Institute of Technology, Newark, NJ 07102, USA; 2 Faculty of Fisheries Technology and Aquatic Resources, Maejo University, Chiang Mai, 50290, Thailand

## Abstract

Fishes have adapted a number of different behaviors to move out of the water, but none have been described as being able to walk on land with a tetrapod-like gait. Here we show that the blind cavefish *Cryptotora thamicola* walks and climbs waterfalls with a salamander-like diagonal-couplets lateral sequence gait and has evolved a robust pelvic girdle that shares morphological features associated with terrestrial vertebrates. In all other fishes, the pelvic bones are suspended in a muscular sling or loosely attached to the pectoral girdle anteriorly. In contrast, the pelvic girdle of *Cryptotora* is a large, broad puboischiadic plate that is joined to the iliac process of a hypertrophied sacral rib; fusion of these bones in tetrapods creates an acetabulum. The vertebral column in the sacral area has large anterior and posterior zygapophyses, transverse processes, and broad neural spines, all of which are associated with terrestrial organisms. The diagonal-couplet lateral sequence gait was accomplished by rotation of the pectoral and pelvic girdles creating a standing wave of the axial body. These findings are significant because they represent the first example of behavioural and morphological adaptation in an extant fish that converges on the tetrapodal walking behaviour and morphology.

The vertebrate transition from finned to limbed appendages in the Devonian period resulted in the earliest records of quadrupedal walking on terra firma. However, fishes, which include more than 35,000 species, have evolved terrestrial locomotion, in one form or another, multiple times. Several extant fishes have a number of morphological and behavioural traits that facilitate moving out of water to escape predation, find food or new habitats, or lay eggs^1^,^2^,^3^. The most simplistic and least anatomically derived method to move across a dry horizontal surface is to undulate or flip the body by modifying the same motor programs that facilitate swimming and escape responses in water, as is seen in eels^4^, killifishes^2^,^5^, and sticklebacks^6^. Some fishes are able to walk underwater using their pectoral fins and pelvic fins (e.g. frogfish^7^); however, in submerged walking the bodyweight is supported by the fluid around the organism. Polypterus^8^, mudskippers^9^,^10^ and walking catfish^11^ move on land via crutching or lateral pushing by the posterior body and tail to rotate forward over their pectoral fins. Lungfish can walk on a horizontal surface using primarily pelvic driven alternating fin movements^12^. Fishes known for vertical climbing, such as Hawaiian waterfall climbing gobies, use either intermittent, rapid axial undulation (*Awaous guamensis* and *Lentipes concolor*) or oral and pelvic suction (*Sicyopterus stimpsoni*)^13^. Notably, none of these fishes walk with a diagonal-couplets lateral sequence gait on land, which has been described as a purely tetrapodal innovation.

The blind cavefish *Cryptotora thamicola* (Cypriniformes: Balitoridae; Fig. 1) can walk up rocks in fast-flowing water and on wet surfaces in air. This fish is a troglobitic hillstream loach endemic to the Tham Maelana and Tham Susa karst formation in northern Thailand. *Cryptotora* are found only in rapids and not in lentic pools^14^. They are commonly observed climbing steep rock surfaces in fast-flowing waterfalls created by basalt or andesite intrusions (Supplementary Video 1)^14^. While it is anecdotally known that these fish can walk, the rare and protected status of these fish has limited research into the functional morphology of their walking behaviour. Here we show that the blind cavefish *Cryptotora thamicola* convergently evolved a diagonal-couplets lateral sequence gait method of walking and shares morphological features typically attributed to the evolution of terrestriality in early tetrapods.

## Results and Discussion

### Pelvic girdle morphology

**Figure 2:**
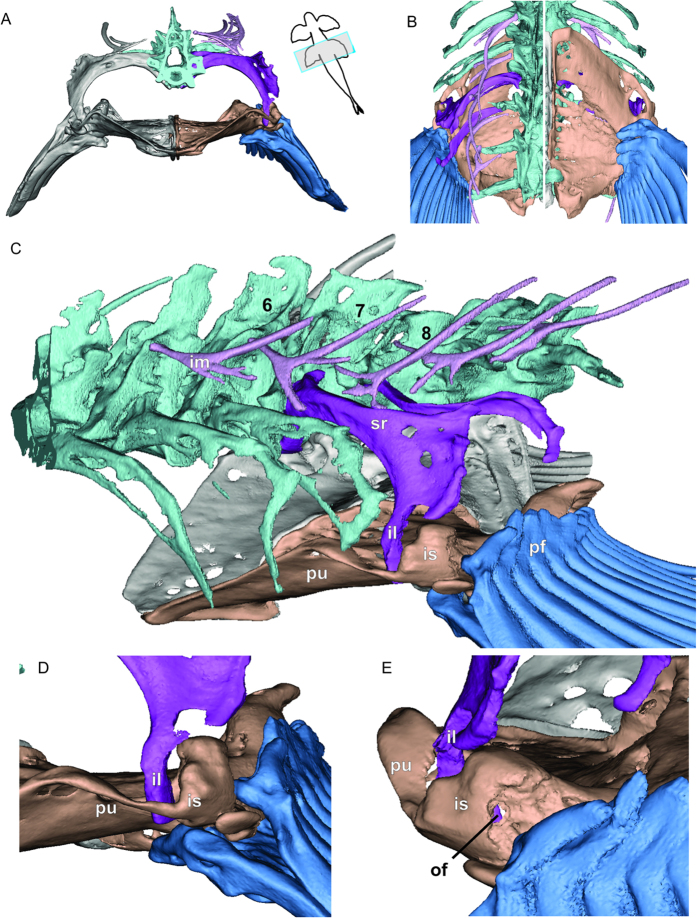
Computed microtomography scan (voxel size = 8 μm) of *Cryptotora thamicola* (47 mm total length). (**A**) transverse view of pelvic girdle, (**B**) dorsal (left) and ventral (right) view pelvic girdle (cranial to top), (**C**) anterolateral view of pelvic girdle. *il*, iliac region (dark purple); *im*, intermuscular bone (light purple); *is*, ischial region of puboischiadic plate (tan); *pf*, pelvic fin (blue); *pu*, pubic region of puboischiadic plate (tan); *sr*, sacral ribs (dark purple). Vertebrae numbers 6,7,8 designate position from skull, (**D**) close-up image of acetabular symphysis, (**E**) close up image of obturator foramen, *of*.

**Figure 3:**
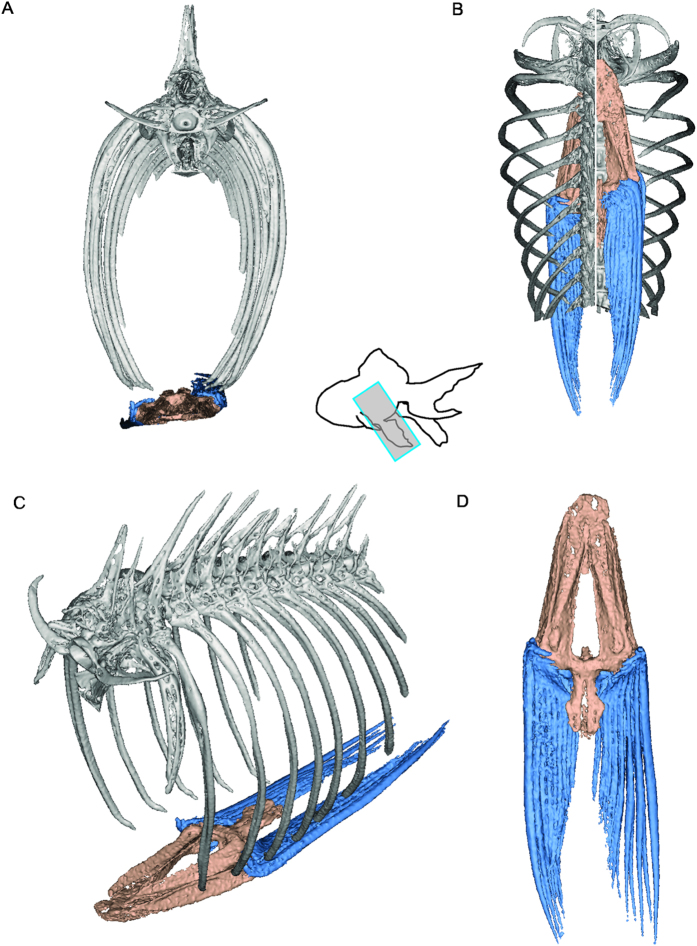
Computed microtomography scan (voxel size = 7 μm) of goldfish *Carassius auratus* pelvis (38 mm total length). (**A**) Transverse view of puboischiadic plate (basipterygium, tan), pelvic fins (blue), and axial skeleton (grey), (**B**) dorsal (left) and ventral (right) view pelvis and axial skeleton (cranial to top), (**C**) anterolateral view of pelvis and axial skeleton. (**D**) dorsal view of pelvis and fins.

We found that *Cryptotora* had a pelvic girdle that is connected to the axial skeleton via fusion with a hypertrophied rib of the seventh vertebra (Fig. 2A–C, Supplementary Video 2). The basipterygium, or puboischiadic plate (*pu*, tan, Fig. 2C), was broad in both anteroposterior and lateral directions and more heavily ossified than is typical of fishes (Fig. 3), with a large concavity for ventral attachment of muscles to the large processes at the base of the fin rays (*pf*, blue, Fig. 2B,C). The heads of the fin rays articulated with the lateral edges of the ischium.

The ribs of *Cryptotora* were broader and extended more laterally than is typical of other fishes; the common goldfish (*Carassius auratus*), also of the order Cypriniformes, is shown for comparison (Figs 2 and 3). Notably, the goldfish pelvis did not articulating with the ribs, but was held in a muscular sling; this is typically the case for all other fishes in which the pelvis is not fused to the pectoral girdle anteriorly. In *Cryptotora*, the first seven ribs were broad and flat with an arch reflecting the dorsoventral compression of the fish body. The seventh rib (*sr*, purple, Fig. 2C) was more heavily calcified than more anterior ribs, which were only calcified around their edges. In addition, the seventh rib had a large, broad, flared process similar in appearance to the iliac crest which supports attachment of large hip flexors in tetrapods. The distal end of the seventh rib (originating on the seventh vertebrae) formed a ventrally oriented iliac process that passed through an arch formed by the pubis and ischium to fuse on the anterolateral aspect of the puboischiadic plate (Fig. 2C). The area of this arch where the puboischiadic plate and iliac process of the sacral rib united was the acetabular symphysis. (Fig. 2D). While extant fishes lack femora, they have historically had the posterior ridge of the pelvis, where the pelvic fins articulate, identified as the area of the acetabulum^15^,^16^. Exctinct fishes *Gooloogongia*, *Eusthenopteron*, and *Panderichthyes* posessed more cup-like acetabula in a similar posterior position, despite lacking limbs^16^,^17^. In contrast, *Cryptotora* had an acetabular symphysis separate from and anterior to the pelvic fin articulation with the puboischiadic plate. Also visible posterior to the acetabular symphysis was the obturator foramen, an opening formed between the ischium and pubis through which blood vessels and nerves may pass (Fig. 2E). The eighth rib did not originate off the vertebral centra as the seventh rib did, but instead originated off a transverse process of the eighth vertebra and extended laterally and hooked anteriorly, which may provide additional area for muscle attachment on the dorsal aspect of the fin.

Based on their positions, we presume that the dorsolateral projections from the vertebral centra are intermuscular bones (*im*, light purple, Fig. 2C) - intramembranous ossifications which form in myosepta as a result of mechanical stress^18^. The intermuscular bone associated with the seventh vertebrae was different than the rest, in that it was attached to the dorsal aspect of the sacral rib and not the vertebral centra. Anterior to the pelvis, the intermuscular bones branched off of the neural arches. If these bones are in fact, not intermuscular, then they are skeletal structures that have not previously been identified in fishes.

The vertebral column of *Cryptotora* shared several features associated with the evolution of terrestriality and the functional need to support the body weight of an organism outside of water: in particular, the wide neural spines, zygapophyses, and transverse processes of *Cryptotora* are features that stabilize the vertebral column in terrestrial vertebrates^19^,^20^. Broad neural spines are typically associated with muscle attachment along the vertebral column^15^. Large transverse processes extended laterally off the vertebral centra and zygapophyses extended in anterior and posterior directions (Fig. 2C). The centra of the sacral vertebrae were tapered ventroposteriorly, resulting in narrowing of the notochord which passed through the center.

**Figure 4:**
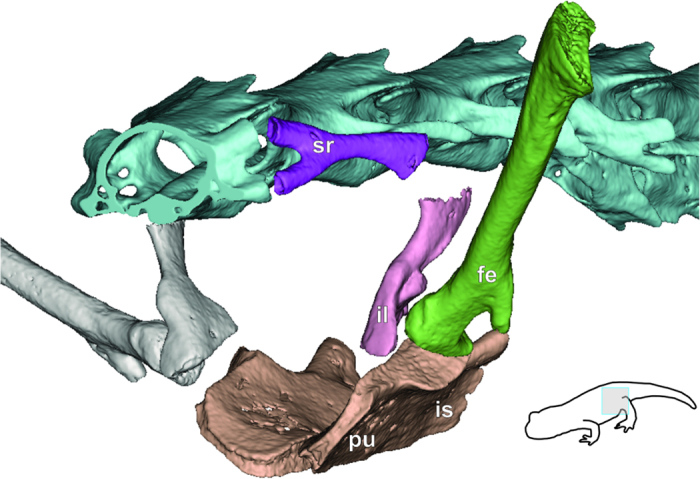
Computed microtomography scan (voxel size = 7 μm) of salamander *Eurycea longicauda* (USNM 543595) pelvis (30.9 mm body length minus tail). Anterolateral view of pelvic girdle. *fe*, femur (green); *il*, iliac bone (pink); *is*, ischial region of puboischiadic plate (tan); *pu*, pubic region of puboischiadic plate (tan); *sr*, sacral rib (dark purple).

While a study of the development of the pelvic girdle in this cavefish has not yet been possible, it appears that structurally, the pelvic girdle of *Cryptotora* converges on tetrapodal morphology that supports muscular attachment and transfer of forces for terrestrial walking^16^,^21^. In other fishes, including those that can walk by crutching or lunging, there is no bony connection between the pelvis and vertebral column and the pelvis is held in place by a muscular sling (Fig. 3)^16^ or fused anteriorly to the pectoral girdle, as in the mudskipper^22^. However, in tetrapods, the ilium originates from the pubis and extends dorsally to fuse with a sacral rib^16^. The incomplete fusion of these two skeletal elements is visible in amphibians (Fig. 4), in which the sacral rib and ilium are joined by cartilage.

### Walking kinematics

*Cryptotora thamicola* walked on rough and smooth wet surfaces while out of water. We observed that *Cryptotora* could walk towards the direction of flowing water (Supplementary Video 3). Hillstream loach relatives of *Cryptotora* in the family Homalopteridae are known to have epidermal ridges and spines to facilitate adhesion to surfaces in fast-flowing water^1^,^23^, but it was not obvious from the μCT scans that these were present on *Cryptotora*.

**Figure 5:**
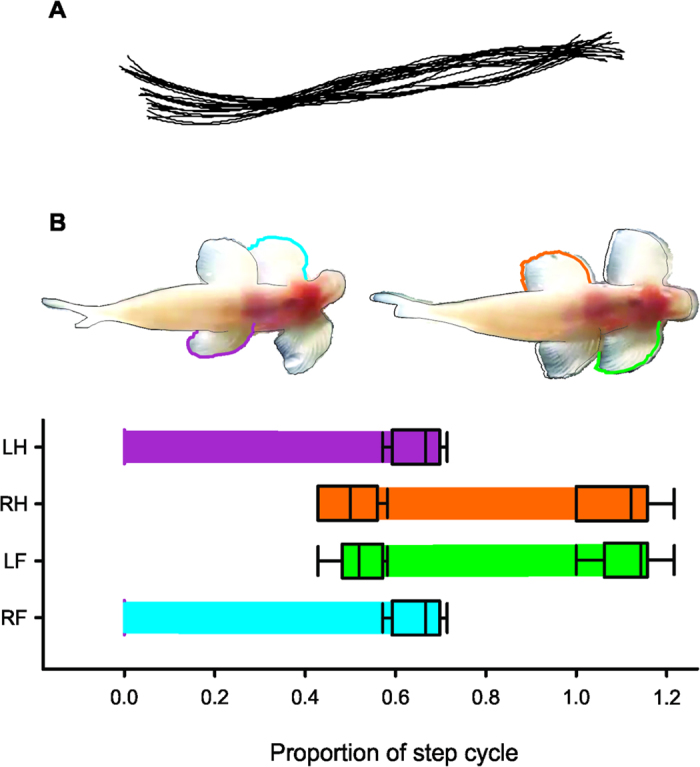
Walking kinematics of *Cryptotora thamicola* climbing a glass surface at a 45 degree angle, ventral view. (**A**) Midline spline curve from five consecutive step cycles, head is to the right and aligned to same position among frames. (**B**) stance phase averaged by proportion of step cycle for right forefin (RF, blue), left forefin (LF, green), right hindfin (RH, orange), and left hindfin (LH, purple). The ends of the bars are calculated as mean ± SE touchdown/liftoff for n = 10 step cycles. Boxes at the ends of bars represent 75% confidence intervals. Time zero was designated as the frame where the LH could be seen pushing away from the glass, which was chosen because this motion was consistently observed to happen in a single frame.

During walking, the body midline of *Cryptotora* (Fig. 5A) moved in a standing wave, and did not follow an undulatory, traveling wave pattern as a swimming fish might use. Swimming fish generally undulate in such a way that little lateral displacement occurs near the trunk and the greatest amplitude of undulation occurs posteriorly, in the tail^24^. All of the fish species that “walk” on land, including mudskippers^9^, lungfish^12^, polypterus^8^, snakeheads, climbing perch^25^, and catfishes, do so by using their tail to push their bodies forward, pivoting over the pectoral fins. In *Cryptotora*, there was no undulation of the tail and body bending occurred primarily between the appendicular girdles, as in salamanders^26^, as the pectoral and pelvic girdles rotate opposite to each other. *Cryptotora* used a diagonal-couplets lateral sequence gait that was observed both in video taken in the fish’s natural habitat (in flow speeds of approximately 60 cm/s) and in a glass aquarium tilted to 45° and 90° (Fig. 5B, Supplementary Video 1, 3). This symmetric alternating gait is typically the most stable for tetrapods because the center of mass is supported by the triangular orientation of the limbs 19,26,27, and was characterised by semi-synchronous movement of the right forefin and lefthindfin (RF-LH) followed by semi-synchronous movement of the left forefin and right hindfin (LF-RH). There was no observable difference in kinematics between sequences collected at 45˚ and 90° inclinations. Walking motion appeared to be primarily driven by rotation of the appendicular girdles relative to the long axis of the fish rather than by protraction and retraction of individual fins; however, further analysis of higher resolution video is necessary to test this.

A step cycle (i.e. stride) was defined as the amount of time between the push off of the left hindfin (LH), which from the ventral view could be seen to rotate the pelvis to the right, until the next LH push off. The gait duty factor was the proportion of the step cycle in which a fin was in contact with the substrate. The duty factor of *Cryptotora* was 65% for each fin with no significant difference among fins (*F*(3,36) = 0.069, *p* = 0.976). This aquatic walking duty cycle falls between the aquatic and terrestrial modes of the salamander *Dicamptodon tenebrosus*, for which the gait pattern duty cycle is approximately 41% while submerged and 77% while walking on a treadmill^26^,^28^.

It has been hypothesized that early tetrapods that could not lift their bodies off the ground would use a crutching^29^ or walking trot gait, in which the body moved with an undulatory wave, and those that could support their weight would perform a lateral sequence walk, resulting in a standing wave of the axial body^19^. *Cryptotora* performs a tetrapodal diagonal-couplets lateral sequence walk with a standing wave but it’s body remains close to the ground, which may be hydrodynamically important when walking in high velocity flow conditions. In reality, the behaviour and morphology of *Cryptotora* are unique as compared to both swimming fishes and walking salamanders and represent a locomotor strategy that may be heavily constrained by their cave environment.

### Evolution of walking in fishes

Tetrapod locomotion began in the water, where the first limbs were used as paddles^30^. A hallmark of the evolution of tetrapods is the regionalisation of vertebral column articulations between neural centra and with the pelvic girdle for support out of the buoyant fluid environment^19^. Indeed, the evolution of the pelvic girdle structure points to development on land^21^; where it is absolutely essential to have a robust mechanical connection between the axial skeleton and the ground to generate upright walking forces^30^. In fishes, both living and extinct, the body is supported by the surrounding fluid; pelvic fins are used primarily for stabilization against roll^31^.

A number of researchers have shown that it is possible to use the biomechanical knowledge of extant vertebrates to attempt to better understand the functional constraints that shaped the origin of tetrapod limbs^32^,^33^,^34^,^35^,^36^. Even though fish bone is anosteocytic, it has been shown to remodel and reinforce skeletal structures in response to mechanical stress^37^. This process is known to cause the formation of intermuscular bones in fishes^15^ and to increase pectoral girdle shape and thickness in polypterus, which walks on land using its pectoral fins^8^. Thus, the enlarged neuropophyses, ribs, and pelvic girdle of *Cryptotora* may have evolved as a result of mechanical stressors from climbing and walking. Developmental plasticity plays an important role in the appearance of complex heritable phenotypic traits^38^,^39^ and extreme environments, like those in caves, are catalysts for the evolution of novel traits. Standen *et al*. hypothesized that environmentally induced phenotypic plasticity may facilitate macroevolutionary change^8^; however, our current data does not allow us discriminate between selection for a robust pelvic girdle specifically or for the plastic response to extreme environmental conditions.

**Figure 6:**
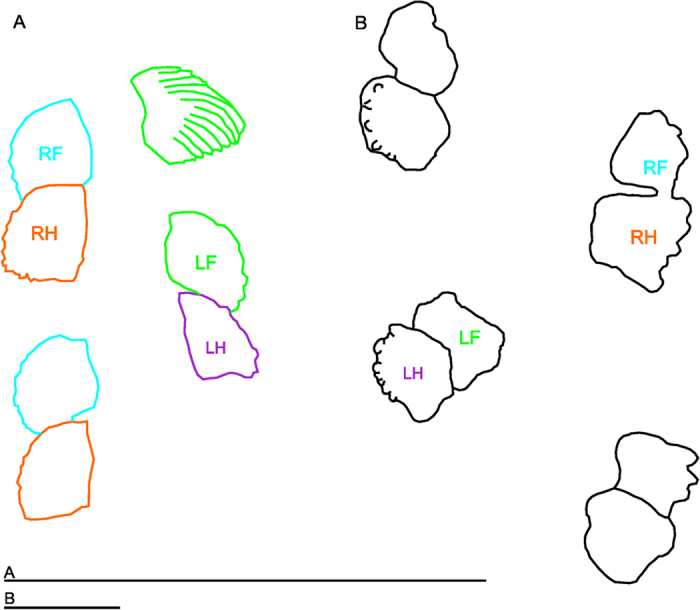
Finprint comparison. (**A**) Tracing of *Cryptotora* fins from ventral view during walking sequence on glass at 45° angle. Final LF (green) fin tracing is shown with fin ray orientations drawn. (**B**) footprint outlines of earliest known tetrapod trackway from the Devonian, redrawn from Warren and Wakefield^40^, rotated to match walking direction of *Cryptotora* (towards top of page), and scaled to similar size (scale bars are 50 mm, bottom).

Interestingly, the fin shape and footfalls of walking *Cryptotora* (Fig. 6A) is similar to the patterns of footprints seen in the oldest known Devonian subaqueous tetrapod trackway (382–358 mya; Fig. 6B) from the Genoa River Beds in New South Wales, Australia^40^. In both cases the pes (hindfin) print overlies the manus (forefin) on the same side^33^,^40^. Also, the six or more digit prints at the edges of the trackway prints match the orientation and position of the distal end of fin rays in walking *Cryptotora*; it is not unreasonable that fin rays digging into a substrate, especially when the fin ray tips are free of webbing as they are in *Cryptotora*, would create a pattern like digits. No known limbed early tetrapods made such tracks^33^,^41^ but they could have been made by a fish that walked like *Cryptotora*. Similar trackways are known from the Valencia Slate Formation in Ireland and these sediments have an earlier date than the presumed origin of tetrapods as indicated by body fossil data^19^. Notably, many Homalopterid fishes are able to push their bodies off the substrate and stand perched on their fins^1^; however, we predict that if *Cryptotora* were to walk on a soft substrate (which is non-native to its cave environment), it would likely leave a body and tail imprint, like that seen with some of the tracks in the Genoa River Bed^40^.

It is, however, crucial to note that *Cryptotora* is not an analogous representative of any early tetrapodamorph described to date. Description of the morphological attributes of early tetrapods that were instrumental in the fin to limb transition included the presence of zygapophyses and large neural spines on vertebrae and a pelvic girdle that was firlmly attached to the axial skeleton; *Cryptotora* convergently evolved these while no other fish has. However, *Cryptotora* obviously lacks digited appendages, which evolved before the pelvic girdle in the fin-to-limb transition.

In the Devonian period, vertebrates gained novel morphological and behavioural traits that facilitated life in a terrestrial environment. Among these, solid-substrate based locomotion, alternating gait by pelvic propulsors, and digit-bearing limbs are considered to be critical adaptations for the emergence of tetrapods^12^,^33^,^42^. Recent works on terrestrial locomotion in fishes have shown that pelvic appendage driven locomotion on a hard surface was possible before the evolution of digited limbs^12^. Our work supports those findings and goes on to show that fish are capable of evolving a robust pelvic girdle that is firmly attached to the vertebral column in the absence of digited limbs; this finding is especially worth considering given the fossils trackways showing tetrapodal locomotion that predate the origin of digited limbs. While there have been multiple cases of secondarily aquatic vertebrates, we only have fossil evidence of one period of time in which vertebrates emerged from an aquatic lifestyle and evolved terrestrial walking behaviour^19^. Future studies of extant fishes that convergently evolved morphological and behavioural features typical of tetrapods will offer a window into understanding the biomechanical constraints that enhanced selection on this complex transformation.

## Experimental Procedures

### Morphological analysis

A paratype specimen of *Cryptotora thamicola* (NIFI 03046, Tham Maelana, Ban Maelana, Tumbon Pangmapha, Pangmapha district, Mae Hong Son Province, Thailand; 47.05 mm total length, TL) was scanned in a Skyscan 1173 computed microtomography (μCT) scanner located at the Advanced Dental Technology Center in Thailand Science Park, Patumthanee Province, Thailand. The scan was performed at 8.8 μm voxel (50 kV, 160 uA) resolution^43^. One common goldfish (*Carassius auratus*; 38 mm TL) and one salamander (*Eurycea longicauda*, USNM 543595; 30.9 mm body length excluding tail) were scanned in a Skyscan 1173 μCT scanner at the molecular imaging center at Rutgers University, New Brunswick, NJ at 6.9 μm voxel resolution. Three-dimensional reconstruction of the μCT scans was performed in Mimics 18 (Materialise USA, Plymouth, MI). Because collection of live *Cryptotora* specimens and dissection of museum specimens is prohibited, all morphological details for this species were extracted through analysis of the high resolution μCT scan. No manipulation of the *Cryptotora* specimen was permitted by the Department of Fisheries in Thailand, including staining with contrast solutions that would allow visualisation of soft tissues by μCT scanning.

### Kinematic analysis

*Cryptotora thamicola* were observed *in situ* at Mae Lana cave, Ban Maelana, Tumbon Pangmapha, Pangmapha district, Mae Hong Son Province, Thailand. Animals are restricted from being removed from the cave and all kinematic video data were obtained at the site the fish were found. Two individuals were carefully scooped into a glass tank for kinematic sequence recording; fourteen walking sequences of 4–9 steps in a straight line at 45° (n = 8) and 90˚ angles (n = 6) were recorded using a Sony HDR-PJ340E camera at 25 fps, which captured 8–10 frames per step cycle. The low sample size in this study reflects the limited access to specimens, and therefore the descriptive kinematics of the walking behaviour presented here should not be treated as a comprehensive kinematic analysis. Fin kinematics were digitised using the DLTdv5 MATLAB 2014a (Mathworks, USA) program developed by T. Hedrick^44^. Cavefish selected for study were released unharmed after approximately 15 minutes of filming. All animals were handled ethically according to institutional animal care and use protocols approved by the New Jersey Intitute of Technology (14–036).

## Additional Information

**How to cite this article**: Flammang, B. E. *et al*. Tetrapod-like pelvic girdle in a walking cavefish. *Sci. Rep*. **6**, 23711; doi: 10.1038/srep23711 (2016).

## Supplementary Material

Supplementary Information

Supplementary Video S1

Supplementary Video S2

Supplementary Video S3

## Figures and Tables

**Figure 1:**
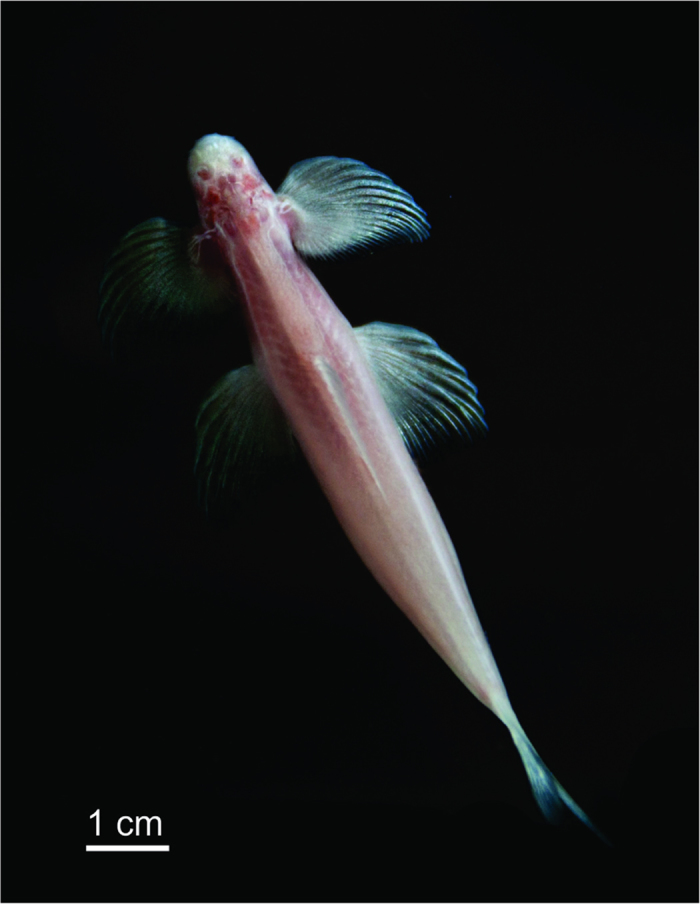
*Cryptotora thamicola*. Dorsal view, resting on the bottom of glass tank. Photo by D.S.
