# Supplementary material for: Tetrapod-like pelvic girdle in a walking cavefish
Source: Sci Rep. 2016 Mar 24;6:23711. doi: 10.1038/srep23711 (PMC4806330; doi:10.1038/srep23711)
Supplement: Supplementary Information [file srep23711-s1.pdf]

Tetrapod-like pelvic girdle in a walking cavefish

Brooke E. Flammang[1]*, Apinun Suvarnaraksha[2], Julie Markiewicz[1], Daphne Soares[1]

[1]Department of Biological Sciences, New Jersey Institute of Technology, Newark, NJ USA 07102

[2] Faculty of Fisheries Technology and Aquatic Resources, Maejo University, Chiangmai, Thailand 50290

*Author for correspondence (flammang@njit.edu)

SUPPLEMENTAL INFORMATION

Supplementary Video 1. Cavefish walking up rock in strong flow. Blind cavefish, *Cryptotora thamicola*, walking up approximately 45˚ incline rock face in strong flow.

Supplementary Video 2. Reconstruction of pelvic girdle from µCT scan. Segmented reconstruction of computed microtomography scan of the pelvic girdle of the blind cavefish, *Cryptotora thamicola*. Color format follows Fig. 2: *dark purple*, sacral rib and iliac process; *tan*, puboischiadic plate; *blue*, pelvic fin.

Supplementary Video 3. Cavefish walking up glass wall. Blind cavefish, *Cryptotora thamicola*, walking up approximately 90˚ incline tank wall in direction of trickling water flow.

The µCT scan slice image files are available via Open Science Framework at dedicated repository link http://osf.io/ahswb
